# Development of a Simple Assay Method for Adenosine Deaminase via Enzymatic Formation of an Inosine-Tb^3+^ Complex

**DOI:** 10.3390/s19122728

**Published:** 2019-06-18

**Authors:** Suji Lee, Heewon Park, Yeongcheol Ki, Hohjai Lee, Min Su Han

**Affiliations:** Department of Chemistry, Gwangju Institute of Science and Technology, 123 Cheomdangwagi-ro, Buk-gu, Gwangju 61005, Korea; jabtneod@gist.ac.kr (S.L.); ynotzzz8@gist.ac.kr (H.P.); YCKi@gist.ac.kr (Y.K.)

**Keywords:** adenosine deaminase, enzyme assay, lanthanide complex, antenna effect

## Abstract

Adenosine deaminase (ADA), which catalyzes the irreversible deamination of adenosine to inosine, is related to various human diseases such as tuberculous peritonitis and leukemia. Therefore, the method used to detect ADA activity and screen the effectiveness of various inhibitor candidates has important implications for the diagnosis treatment for various human diseases. A simple and rapid assay method for ADA, based on the enzymatic formation of a luminescent lanthanide complex, is proposed in this study. Inosine, an enzymatic product of ADA with stronger sensitization efficiency for Tb^3+^ than adenosine, produced a strong luminescence by forming an inosine-Tb^3+^ complex, and it enabled the direct monitoring of ADA activity in real-time. By introducing only Tb^3+^ to adenosine and ADA in the buffer, the enhancement of luminescence enabled the detection of a low concentration of ADA (detection limit 1.6 U/L). Moreover, this method could accurately determine the inhibition efficiency (IC_50_) of the known ADA inhibitor, erhythro-9-(2-hydroxy-3-nonyl)adenine (EHNA), and the inhibition of ADA could be confirmed by the naked eye. Considering its simplicity, this assay could be extended to the high-throughput screening of various ADA inhibitor candidates.

## 1. Introduction

Adenosine deaminase, referred to as ADA (EC 3.5.4.4), is a key enzyme in purine metabolism, which catalyzes the irreversible deamination of adenosine (deoxyadenosine) to inosine (deoxyinosine) [1,2]. ADA is distributed throughout all human tissues, and its abnormal level is related to various human diseases. Accumulation of ADA substrate deoxyadenosine, due to the extremely low activity of ADA, causes the inhibition of DNA synthesis by the sequential inhibition of ribonucleotide reductase [3,4]. In particular, it is well known that the genetic deficiency of ADA leads to severe combined immunodeficiency disease (SCID), which causes serious infections and chronic diarrhea [5,6,7,8]. In contrast, the high level of ADA activity may result in several serious diseases such as tuberculous peritonitis and leukemia [9,10,11,12]. Thus, inhibiting the high activity of ADA can be an effective measure to treat several lymphoproliferative disorders. In addition, inhibition of ADA prevents postischemic heart injury by blocking of sequential substrate formation for xanthine oxidase which generates reactive oxygen species in the heart [13], and recent studies have reported the potential of inhibiting ADA activity during antitumor therapy, revealing that it can suppress the progression of breast cancer cells [14]. Therefore, detecting ADA activity has important implications in the diagnosis and rapid verification of the effectiveness of candidate drugs for various human diseases. 

Traditionally, spectrometric assay of ADA measured ADA activity based on detecting the change in intrinsic absorbance of uric acid that is converted from inosine via sequential enzyme cascade reaction using the combination of nucleoside phosphorylase and xanthine oxidase [15]. Other methods were based on the color change developed by the chemical reaction of ammonia or uric acid, which is the product of these enzymatic cascade reactions, with colorimetric oxidizing agent [16]. Nevertheless, these methods have certain limitations as described further. Initially, they require extra enzymes for the enzyme-coupled reaction or these extra enzymes and colorimetric agent. Additionally, they cannot detect ADA activity in real-time and this is time-consuming due to the enzyme-coupled reaction and chemical reactions with the colorimetric agents. Recently, several fluorometric, colorimetric, and electrochemical ADA assay methods have been developed using adenosine aptamer [17,18,19,20,21,22]. These methods use real-time assay and do not require extra enzymes for the enzyme-coupled reaction; however, certain issues persist such as the complicated system considering the conformational change of aptamer by competitive binding of substrate and product, and the stability of the aptamer conjugate. Thus, the development of a simpler method, which can be applied in real-time detection of ADA activity and rapid screening of various ADA inhibitor candidates, is still necessary.

**Scheme 1:**
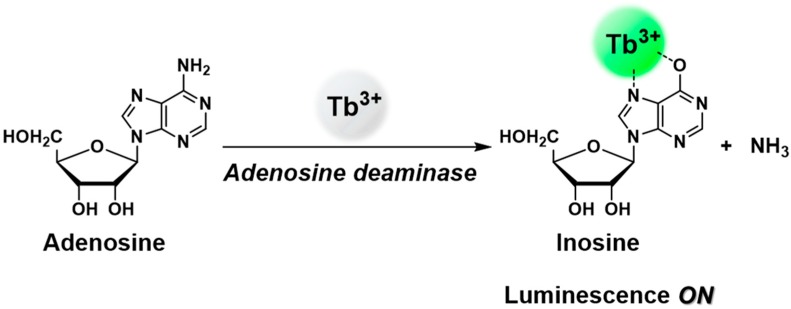
Schematic illustration of adenosine deaminase assay via enzymatic formation of an inosine-Tb^3+^ complex.

A lanthanide complex has been widely used as a sensitive probe for various targets such as anions, metal ions, and enzymes due to the advantage of long-lived narrow emission and large Stokes shift compared to most organic fluorophores [23,24,25,26,27,28,29]. Furthermore, various studies about ligands for lanthanide ions have been investigated as a sensitizer for developing a lanthanide complex with strong luminescence [30,31,32,33,34]. Among these ligands, it is observed that nucleosides with ribose hydroxyl groups strongly bind to the lanthanide ion; meanwhile, adenosine reveals weaker complexation than inosine because adenosine only has a nitrogen donor in its nucleobase [35,36]. Considering this result, we envisioned that adenosine and inosine could be distinguished by their different sensitization efficiencies for the lanthanide ion; this difference resulted in a change in luminescence intensity via the enzymatic formation of an inosine–lanthanide complex. Herein, we developed a simple and direct ADA assay method using the different sensitization efficiencies of adenosine and inosine for lanthanide ion. Inosine produced from the ADA reaction of adenosine bound to the lanthanide ion and the inosine–lanthanide complex produced strong luminescence (Scheme 1). Based on the enzymatic formation of the inosine-Tb^3+^ complex, this method could measure the ADA activity in real-time; moreover, it could be applied for screening the inhibition efficiency of potent ADA inhibitors.

## 2. Materials and Methods

### 2.1. Materials and Instrumentation

For the enzyme assay, ADA (from calf intestine) and erythro-9-(2-hydroxy-3-nonyl)adenine (EHNA) were purchased from Sigma-Aldrich. Adenosine, inosine, and terbium(III) chloride hexahydrate (TbCl_3_·6H_2_O) were purchased from Alfa Aesar and all reagents were used without further purification.

Luminescence spectra were obtained via Agilent Cary Eclipse Fluorescence Spectrophotometer (Agilent, Santa Clara, CA, USA). FT-IR spectra were obtained using a Thermo Scientific Nicolet iS10 FT-IR spectrometer (Thermo Fisher Scientific, Waltham, MA, USA). NMR spectra were recorded using a JEOL (400 MHz) NMR spectrometer (JEOL Ltd., Tokyo, Japan).

### 2.2. Optimization of Condition for Discrimination Between Adenosine and Inosine

Various buffers (HEPES or Tris, 20 mM) with different pH (pH 7.0, 7.4, and 8.0) were added to the solution containing ligand (adenosine or inosine, 50 μM) and TbCl_3_·6H_2_O (1 mM). Without further incubation, the phosphorescence intensity at 545 nm was recorded. Control sample comprised only TbCl_3_·6H_2_O (1 mM) in the buffer solution (HEPES or Tris, 20 mM).

Each of adenosine and inosine with different amount (0, 10, 20, 30, 40, 50 μM) were added to the solution containing TbCl_3_·6H_2_O (1 mM) and HEPES buffer (pH 8.0, 20 mM). Without further incubation, the phosphorescence intensity at 545 nm was recorded.

All samples were adjusted to a final volume of 1 mL and all measurements were carried out in 1 cm quartz cell at 25 °C and were repeated thrice, respectively.

### 2.3. Model study of Enzymatic Antenna Formation

The sample containing TbCl_3_·6H_2_O (1 mM) and different ratios of [inosine]/([inosine] + [adenosine]) (0, 10, 20, 30, 40, 50, 60, 70, 80, 90, 100%) was prepared with fixed concentration of [inosine] + [adenosine] = 50 μM. Without further incubation, the phosphorescence intensity at 545 nm was recorded.

### 2.4. Identification of Chemical Structure of Inosine-Tb^3+^ Complex by FT-IR and ^1^H NMR Spectroscopy

To prepare the inosine-Tb^3+^ complex, solution of inosine (10 mM) was mixed with different equivalents of TbCl_3_·6H_2_O (10, 40 mM) and the mixture was lyophilized. Then, IR spectra (using KBr pellets) were taken using an FT-IR spectrometer.

Inosine (5 mM) in D_2_O with different equivalents of TbCl_3_·6H_2_O (2.5, 5, 10, 20 mM) in D_2_O were mixed. Then, NMR spectra were recorded using an NMR spectrometer.

### 2.5. Kinetic Measurement of ADA Enzyme Reaction

Various amounts of ADA (2, 3, 4, 5, 6, 7, 8, 9, 10 mU/mL) were added to the solution containing TbCl_3_·6H_2_O (1 mM) and adenosine (50 μM) in HEPES buffer (pH 8.0, 20 mM). Thereafter, the phosphorescence intensity at 545 nm of sample was recorded for 60 min in 1 min cycle. To get the quantitative relationship between enzymatic conversion and phosphorescence intensity, a calibration curve was obtained from the result of the model study of enzymatic antenna formation.

### 2.6. Detection of ADA Inhibition by EHNA

Various concentrations of EHNA (5, 25, 50, 80, 100, 150, 200, 250 nM) with ADA (0.2 U/mL) were preincubated for 50 min. Thereafter, the phosphorescence intensity at 545 nm of sample containing inhibitors, ADA, TbCl_3_·6H_2_O (1 mM), and adenosine (10 μM) in HEPES buffer (pH 8.0, 20 mM) was recorded immediately for 40 min in 1 min cycle. For calculating the half maximal inhibitory concentration (IC_50_), inhibition efficiency (IE) was defined as:

(IE) = [Δ(P.I.)_(no inhibitor)_ − Δ(P.I.)_(inhibitor)_]/Δ(P.I.)_(no inhibitor)_

where Δ(P.I.) indicates the phosphorescence intensity change at 545 nm in the selected duration.

## 3. Results and Discussion

### 3.1. Optimization of ADA Assay Condition Using Lanthanide Ion

**Figure 1:**
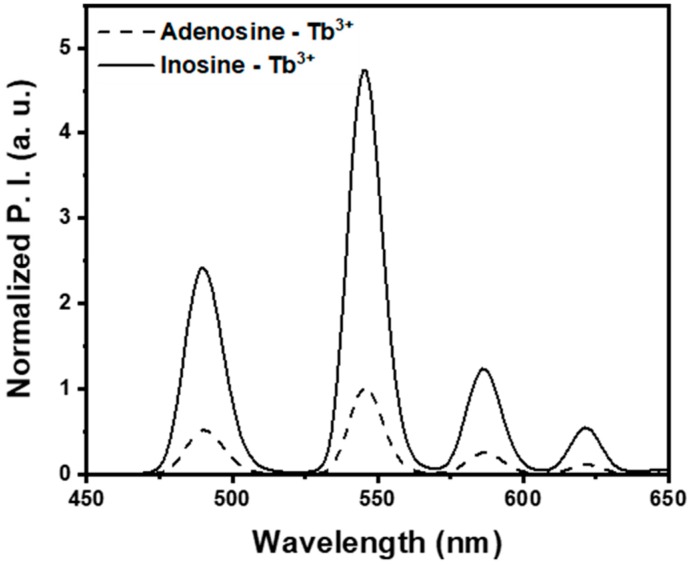
Comparison of luminescence of Tb^3+^ (1 mM)-nucleoside (adenosine or inosine, 50 μM) complex in HEPES buffer condition (pH 8.0; HEPES, 20 mM). λ_ex_ = 260 nm.

First, we screened the change in the luminescence of the Tb^3+^ complex with adenosine and inosine under various buffer conditions to verify whether the discrimination of adenosine and inosine were enabled based on their different antenna effect for the lanthanide complex. As expected, luminescence enhancement occurred in the inosine-Tb^3+^ complex under a basic condition, whereas adenosine indicated no significant change in luminescence in the presence of Tb^3+^ (Figure S1). This indicated that inosine had stronger sensitization efficiency as a Tb^3+^ antenna than adenosine because inosine contains a hard, negatively charged oxygen atom and adenosine only contains nitrogen donors. In addition, inosine-Tb^3+^ complex showed phosphorescence with a lifetime of above 20 μs (Figure S2) [37]. The difference between luminescence intensities between inosine and adenosine with Tb^3+^ was evident under HEPES buffer condition (Figure 1), wherein the HEPES buffer was optimized to a slightly basic pH of 8.

**Figure 2:**
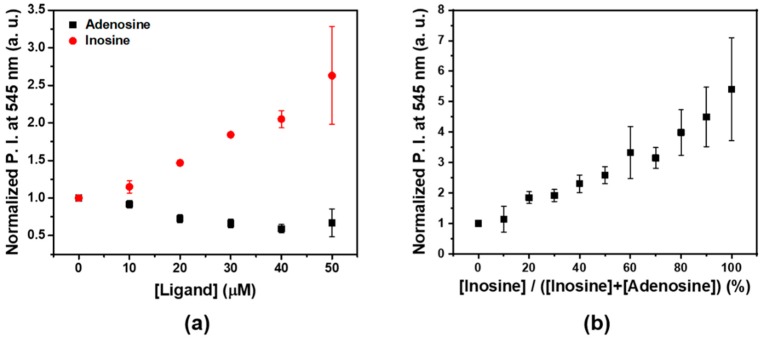
(**a**) Phosphorescence intensities at 545 nm of Tb^3+^ (1 mM)-nucleoside (adenosine or inosine) complex upon addition of various concentrations of nucleoside. (**b**) Phosphorescence intensities at 545 nm of Tb^3+^ (1 mM)-nucleoside complex in presence of various ratios of inosine/adenosine. λ_ex_ = 260 nm.

As presented in Figure 2a, luminescence of Tb^3+^ complex was linearly related to the concentrations of inosine. The luminescence of Tb^3+^ complex increased immediately with addition of inosine to the solution of Tb^3+^ in buffer condition. Thereafter, the luminescence intensity was kept constant for 1 h (Figure S3). In presence of both adenosine and inosine with Tb^3+^, the luminescence of Tb^3+^ linearly increased as the ratio of inosine increased, as in an enzymatic reaction in which the concentration of product increased and that of adenosine decreased simultaneously (Figure 2b).

**Figure 3:**
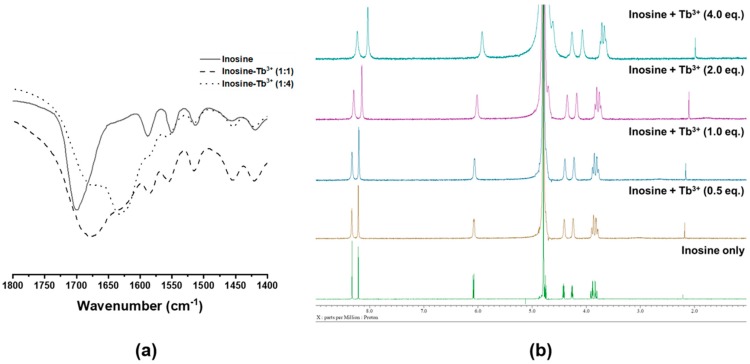
(**a**) IR spectra in the 1400–1800 cm^−1^ region of inosine in absence and presence of Tb^3+^. (**b**) ^1^H NMR profile of inosine free (5 mM) and with different Tb^3+^ equivalents.

To investigate the chemical structure of this luminescent inosine-Tb^3+^ complex, the complex was identified by FT-IR and ^1^H NMR spectroscopy. In the IR spectrum (Figure 3a), C=O stretching band (~1700 cm^−1^) of inosine was shifted to ~1625 cm^−1^ when Tb^3+^ was added, indicating an interaction of Tb^3+^ with oxygen included in hypoxanthine of inosine [38]. In ^1^H NMR spectrum (Figure 3b), the peak of aromatic protons of inosine (δ 8.163, 8.146) was shifted to upfield and its shape was changed. Based on these results and the previous report [39], the proposed chemical structure for inosine-Tb^3+^ complex was accorded to the complex shown in Scheme 1.

### 3.2. Kinetic Measurement of ADA Enzyme Reaction

Based on the result, we anticipated that the antenna effect of inosine for luminescent Tb^3+^ complex could be applicable in monitoring the ADA activity (which converts adenosine into inosine) in real-time. To confirm this, we prepared the cocktail of adenosine and Tb^3+^, and ADA was added to this solution; thereafter, the luminescence change was recorded in real-time.

**Figure 4:**
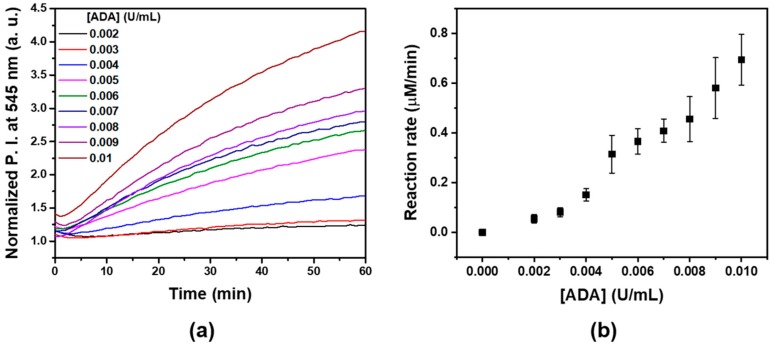
(**a**) Change in the luminescence intensities of a sample containing adenosine (50 μM) and Tb^3+^ (1 mM) with various concentrations of ADA in buffer condition (pH 8; HEPES, 20 mM). (**b**) Plot of the reaction rate of ADA versus various concentrations of ADA in a combination of adenosine (50 μM) and Tb^3+^ (1 mM) in buffer condition (pH 8; HEPES, 20 mM). λ_ex_ = 260 nm.

As presented in Figure 4a, luminescence intensity of Tb^3+^ complex continued to increase as the enzymatic reaction proceeded. To investigate the kinetic behavior of ADA, we measured the luminescence intensities of the cocktail of adenosine and Tb^3+^ with various concentrations of ADA. The reaction rate of ADA was determined based on the change in the luminescence intensity at 545 nm, wherein the rate exhibited linear relationship with ADA concentration (Figure 4b). The detection limit of 1.6 U/L ADA was obtained, which is comparable to other complicated ADA assay methods that use aptasensors or nanomaterials. Thus, these results revealed that the introduction of only Tb^3+^ to ADA and adenosine enabled the simple and direct detection of ADA activity with high sensitivity in real-time based on the enzymatic formation of inosine-Tb^3+^ complex with strong luminescence. Furthermore, we also tried to detect ADA activity in diluted plasma to evaluate the feasibility of our method under biological fluid. As we were concerned about the interference from the unwanted binding of Tb^3+^ to other components such as serum proteins [40], the blank signal of Tb^3+^ in diluted serum was enhanced and it was difficult to detect low concentration of ADA (Figure S4).

### 3.3. Determination of Inhibition Efficiency of ADA Inhibitor

**Figure 5:**
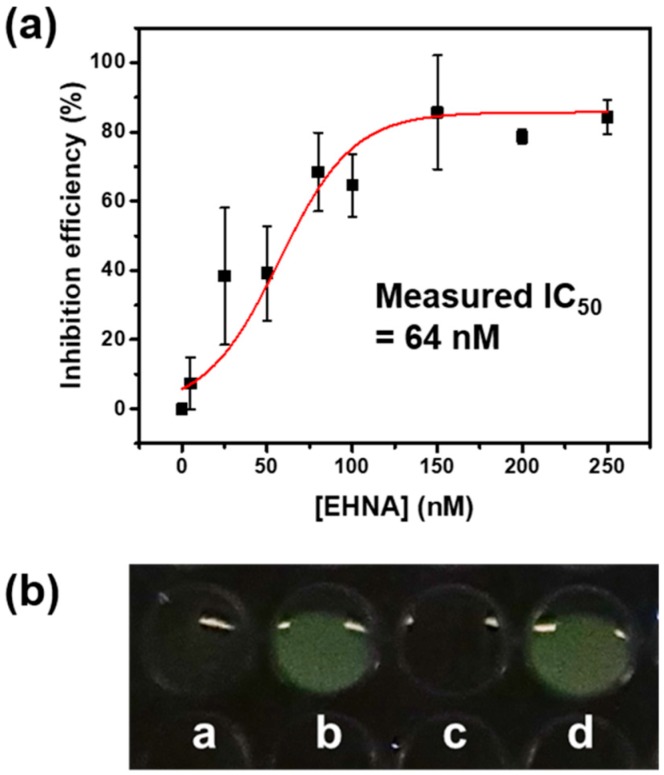
(**a**) Plot of inhibition efficiency of EHNA versus various inhibitor concentrations, condition: adenosine 50 μM, Tb^3+^ 1 mM, ADA 0.2 U/mL in buffer condition (pH 8; HEPES, 20 mM), λ_ex_ = 260 nm. (**b**) Photograph taken under a hand-held UV lamp (254 nm) after 20 min, a: adenosine (1 mM) + Tb^3+^ (20 mM) b: a + ADA (0.2 U/mL), c: b + EHNA (10 μM), d: b + theobromine (10 μM).

To explore the feasibility of this assay method for screening the inhibition efficiency of various ADA inhibitor candidates, we chose EHNA, which is a well-known potent ADA inhibitor [2], as the model inhibitor. After incubating various concentrations of EHNA and ADA, the inhibition efficiency of EHNA depending on the different inhibitor concentrations was measured. As predicted, the activity of ADA was inhibited by EHNA and the inhibition was enhanced as the concentration of EHNA increased. From the dose-response curve for EHNA (Figure 5a), the measured IC_50_ of EHNA was 64 nM and it was in accordance with the literature value [17]. This result demonstrated that this simple assay method using the introduction of Tb^3+^ to ADA and adenosine could screen the inhibition efficiency of ADA inhibitors.

Furthermore, the inhibition of ADA activity and relative inhibition efficiency of various ADA inhibitors could be confirmed by the naked eye via observing the change of green luminescence of the assay solution containing ADA, inhibitor, adenosine, and Tb^3+^ as presented in Figure 5b. After treating 10 μM of ADA inhibitors, we confirmed that EHNA (*K_i_* = 1.6 nM) completely inhibited the ADA activity and had stronger inhibition efficiency than that of theobromine (*K_i_* = 311 μM) [2,41]. Considering its simplicity and rapid speed, it could be applicable to high-throughput screening of various ADA inhibitor candidates in developing a potent ADA inhibitor as therapeutic agent.

## 4. Conclusions

We developed a simple and direct assay method for ADA activity based on the antenna effect of inosine by the enzymatic formation of an inosine-Tb^3+^ complex. Adenosine and inosine had different sensitization efficiencies for Tb^3+^ as antennae, resulting in distinct luminescence of the lanthanide complex. Insertion of inosine to Tb^3+^ under buffer condition remarkably enhanced the luminescence intensity of the Tb^3+^ complex, whereas adenosine had no influence on the luminescence of Tb^3+^ complex. Based on these results, the ADA activity could be monitored in real-time with a combination of adenosine and Tb^3+^; luminescence intensity of the assay solution containing adenosine, Tb^3+^, and ADA increased simultaneously as the enzymatic reaction proceeded. The obtained detection limit for ADA was quite low (1.6 U/L), and was comparable to the previously reported assay method that included a complicated system. Furthermore, it could be used for screening the relative inhibition efficiency of ADA inhibitors by the naked eye, and to measure the IC_50_ of the known ADA inhibitor EHNA (measured IC_50_ = 64 nM) accurately. By the introduction of only a lanthanide ion in the enzymatic reaction, a simple assay for ADA activity and rapid screening of ADA inhibitors was enabled, which might be used as a high-throughput screening method for ADA inhibitor candidates for treating various human diseases.

## Supplementary Materials

The following are available online at https://www.mdpi.com/1424-8220/19/12/2728/s1, Figure S1: Result of buffer screening for optimization of condition for discrimination between adenosine and inosine, Figure S2: Lifetime measurement for luminescence of inosine-Tb^3+^ complex, Figure S3: Time-dependency of luminescence intensity of inosine-Tb^3+^ complex, Figure S4: Confirmation of feasibility of assay method in diluted serum sample.
