# Supplementary material for: Development of a Simple Assay Method for Adenosine Deaminase via Enzymatic Formation of an Inosine-Tb3+ Complex
Source: Sensors (Basel). 2019 Jun 18;19(12):2728. doi: 10.3390/s19122728 (PMC6631010; doi:10.3390/s19122728)
Supplement: Supplementary file 1 [file sensors-19-02728-s001.pdf]

Supplementary data

Development of a Simple Assay Method for Adenosine Deaminase via Enzymatic Formation of an Inosine-Tb^{3+} Complex

Suji Lee †, Heewon Park †, Yeongcheol Ki, Hohjai Lee * and Min Su Han *

Department of Chemistry, Gwangju Institute of Science and Technology, 123 Cheomdangwagi-ro, Buk-gu, Gwangju 61005, Korea; jabtneod@gist.ac.kr (S.L.); ynotzzz8@gist.ac.kr (H.P.); YCKi@gist.ac.kr (Y.K.)

* Correspondence: hohjai@gist.ac.kr (H.L.); happyhan@gist.ac.kr (M.S.H.)

† These authors contributed equally to the work in this paper.

Contents

1. Result of buffer screening for optimization of condition for discrimination between adenosine and inosine

2. Lifetime measurement for luminescence of inosine-Tb^{3+} complex

3. Time-dependency of luminescence intensity of inosine-Tb^{3+} complex

4. Confirmation of feasibility of assay method in diluted serum sample

1. Result of Buffer Screening for Optimization of Condition for Discrimination between Adenosine and Inosine

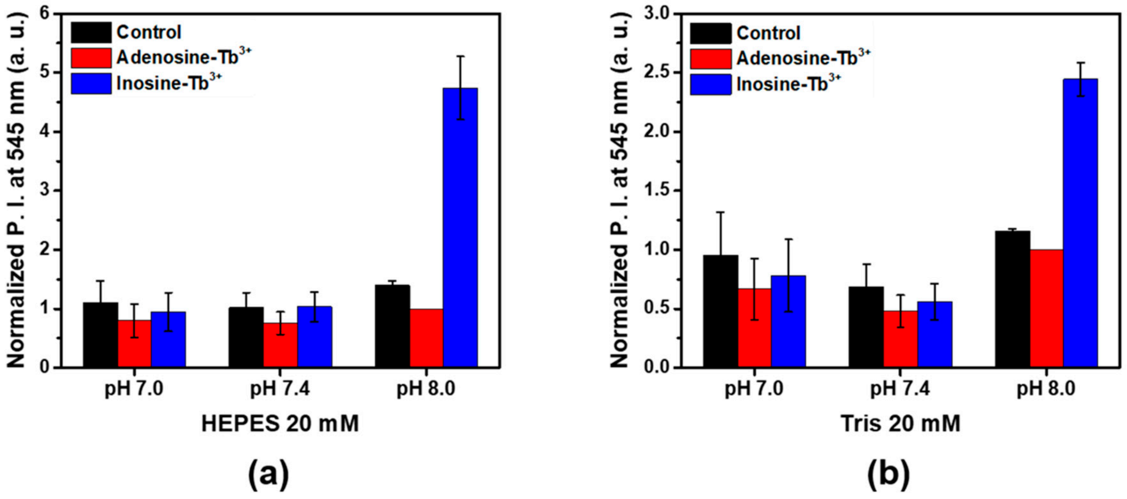

Figure S1. Comparison of luminescence intensities of adenosine- and inosine-Tb^{3+} complexes under

different buffer conditions; (**a**) HEPES 20 mM, (**b**) Tris 20 mM. λ_{ex} = 260 nm.

2. Lifetime Measurement for Luminescence of Inosine-Tb^{3+} Complex

For the luminescence lifetime measurement, 2 mM $TbCl_3$ and 100 μM inosine were mixed in 20 mM HEPES buffer at pH 8. The sample was contained in a quartz cuvette and placed in a cuvette holder with four-way openings (CVH100, Thorlabs). 266-nm laser pulses generated by the 4th harmonics generator in a Nd:YAG laser system (Q-smart, Quantel Laser, 7-nsec pulsewidth, 10-Hz repetition rate) was introduced to the sample cuvette for excitation. The excitation laser power was kept as low as 5 mW. The luminescence from the sample was collected and fed to a PMT (H11526, Hamamatsu) through a liquid light guide (LLG0538, Thorlabs) that was plugged on the cuvette holder along the optical axis perpendicular to the excitation laser pulse propagation. The signal from the PMT was recorded by a digital oscilloscope (TDS2024C, Tektronix). A 532-nm notch filter and a bandpass filter (centered at 540 nm, 25-nm FWHM) were placed in front of the PMT to remove any 532 nm residuals from the Nd:YAG laser system and to allow only Tb^{3+} luminescence at 545 nm. A tri-exponential model was used to fit the luminescence decay curve:

$$I = A_1 e^{(-\frac{t}{\tau_1})} + A_2 e^{(-\frac{t}{\tau_2})} + A_3 e^{(-\frac{t}{\tau_3})} + I_0$$

where I is the luminescence intensity, I_0 is the offset, t is the delay time, A's are the amplitudes of the exponentials, τ's are the lifetimes in microsecond. The averaged lifetime was calculated as follows:

$$\tau_{avg} = \frac{A_1}{A} \times \tau_1 + \frac{A_2}{A} \times \tau_2 + \frac{A_3}{A} \times \tau_3, \text{ where } A = A_1 + A_2 + A_3$$

Fitting result is that A_1= 0.0653, A_2= 0.4461, A_3= 0.4886, τ_1= 108.86 μsec, τ_2= 5.87 μsec, τ_3= 21.39 μsec, and τ_{avg} = 20.17 μsec. The initial 100 ns of the decay curve was ignored for the fitting due to contamination with auto-fluorescence from the quartz cuvette that contains the sample.

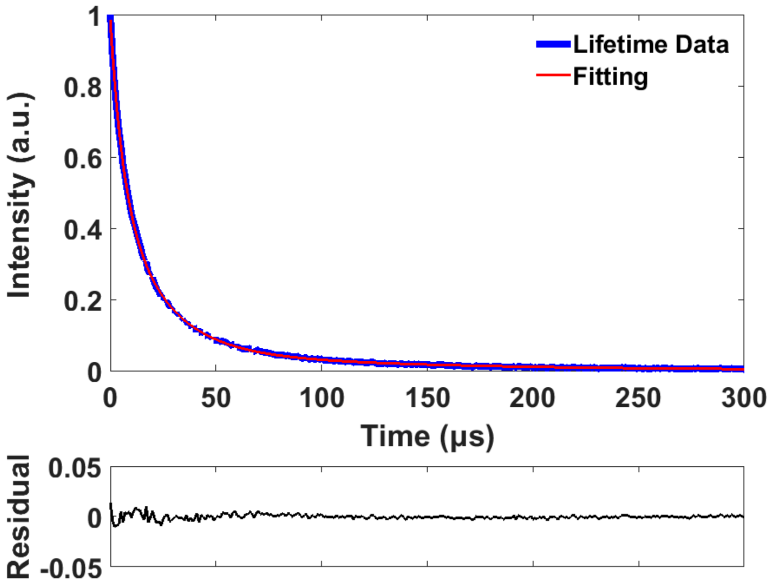

Figure S2. Upper panel: the luminescence lifetime decay curve (blue line) of inosine-Tb^{3+} complex and its tri-exponential fitting result (red line). The averaged lifetime is 20.17 μsec. **Lower panel**: the residual signal after the fitting.

3. Time-Dependency of Luminescence Intensity of Inosine-Tb^{3+} Complex

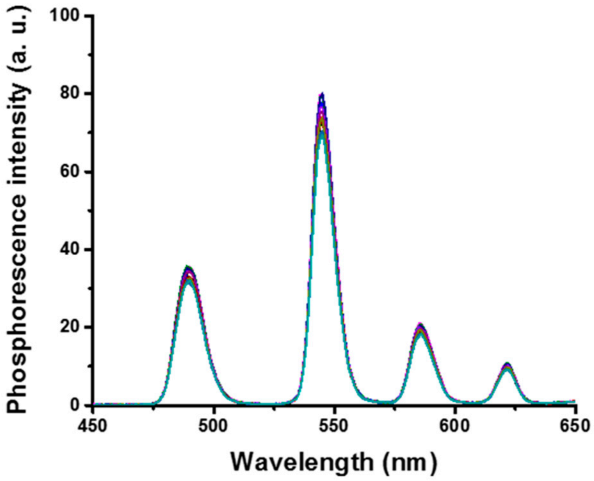

Figure S3. Monitoring of luminescence intensity of inosine (50 μM)-Tb^{3+} (1 mM) complex under a HEPES buffer condition (pH 8; HEPES, 20 mM) for 60 min. λ_{ex} = 260 nm.

4. Confirmation of Feasibility of Assay Method in Diluted Serum Sample

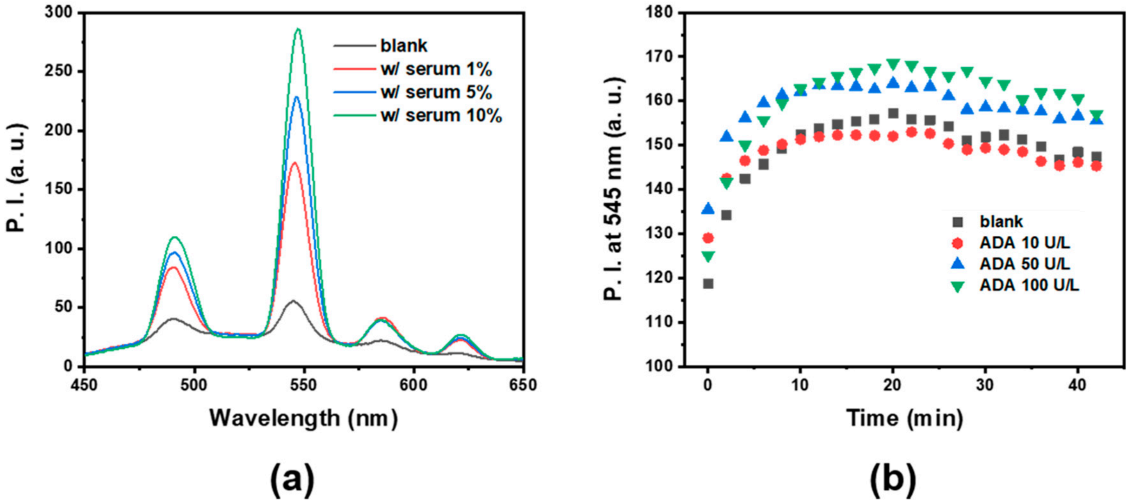

Figure S4. (**a**) Phosphorescence intensities of Tb^{3+} (1 mM) with and without diluted human serum, (**b**) change of phosphorescence intensities at 545 nm of a sample containing adenosine (50 μM) and Tb^{3+} (1 mM) with various concentrations of spiked ADA in presence of 1% human serum. λ_{ex} = 260 nm.
